# Does Jacobson’s relaxation technique reduce consumption of psychotropic and analgesic drugs in cancer patients? A multicenter pre–post intervention study

**DOI:** 10.1186/s12906-018-2200-2

**Published:** 2018-05-02

**Authors:** Paula Parás-Bravo, Cristina Alonso-Blanco, María Paz-Zulueta, Domingo Palacios-Ceña, Carmen María Sarabia-Cobo, Manuel Herrero-Montes, Ester Boixadera-Planas, César Fernández-de-las-Peñas

**Affiliations:** 1 Department of Nursing, Faculty of Nursing, University of Cantabria, Avda.Valdecilla s/n CP, 39008 Santander, Cantabria Spain; 2 Department of Physical Therapy, Occupational Therapy, Rehabilitation, and Physical Medicine, University Rey Juan Carlos, Alcorcón, Spain; 3 Servei d’Estadística Aplicada of the UAB, Autonomous University of Barcelona, Barcelona, Spain; 4 Grupo Enfermería IDIVAL, Santander, Cantabria España

**Keywords:** Psychotropic drugs, Analgesics, Relaxation therapy, Cancer patients, Learning, Complementary therapies

## Abstract

**Background:**

Cancer patients often suffer from emotional distress as a result of the oncological process. The purpose of our study was to determine whether practice of Jacobson’s relaxation technique reduced consumption of psychotropic and analgesic drugs in a sample of cancer patients.

**Methods:**

This was a multicenter pre–post intervention design. Participants were 272 patients aged over 18 years attending 10 Spanish public hospitals with oncological pathologies and anxiety symptoms. The intervention consisted of a protocol of abbreviated progressive muscle relaxation training developed by Bernstein and Borkovec. This was followed up by telephone calls over a 1-month period. The intervention was performed between November 2014 and October 2015. Sociodemographic variables related to the oncological process, mental health variables, and intervention characteristics were measured.

**Results:**

A reduction in the consumption of psychotropic and analgesic drugs was observed throughout the follow-up period. Improvement was observed throughout the 4-week follow-up for all the parameters assessed: anxiety, relaxation, concentration, and mastery of the relaxation technique.

**Conclusions:**

The practice of abbreviated Jacobson’s relaxation technique can help to decrease the consumption of psychotropic and analgesic drugs. Patients experienced positive changes in all the evaluated parameters, at least during the 1-month follow-up. To confirm these findings, additional long-term studies are needed that include control groups.

**Trial registration:**

ISRCTN 81335752, DOI 10.1186/ISRCTN81335752 17. Date of registration: 22/11/2016 (retrospectively registered).

## Background

It has been estimated that between 20% and 50% of patients with cancer experience pain [1] and this estimate increases to up to 90% when the illness is very advanced [2]. Furthermore, approximately 30% of patients experience emotional distress during the course of treatment [3, 4].

The management of oncological pain is particularly difficult and although different recommendations exist, there is currently no clear consensus. The World Health Organization, the American Cancer Society, and the European Society for Medical Oncology mention in their guidelines on managing cancer pain the importance of medications (aside from analgesic treatment) such as psychotropic drugs, although they stipulate that these should be taken only for pain that may otherwise be unmanageable or difficult to control [5–7]. Despite following these recommendations, a high percentage of patients cannot control their pain. A literature review by Deandrea et al. [8] reported that approximately 43.4% are unable to control their pain and some sources suggest that this figure may be as high as 80%.

Cancer patients often experience emotional distress, such as anxiety and depression, and may consume psychotropic drugs to manage both pain and emotional symptoms [9–11].

Previous studies indicate that the consumption of psychotropic drugs, especially anxiolytics and antidepressants, is high among cancer patients. Syrowatka et al. [12] reported a consumption of 50.6% of anxiolytics in women with breast cancer. Barry et al. [13] found that 51% of patients with metastasis cancer took anxiolytics for pain during the active treatment phase. The consumption of antidepressants has been reported as between 10% and 64% [12–14]. Thus, research indicates that a large number of patients consume three or more psychotropic drugs [13].

It is clear, therefore, that consumption of a combination of analgesic and psychotropic drugs is common in cancer patients. Indeed, Kierner et al. [15] reported that the prevalence of consumption of psychotropic drugs and analgesics was as high as 75% and 90%, respectively, during the final illness phases. These results suggest that consumption increases with the evolution of the illness.

Polypharmacy in oncological patients is therefore a problem. In addition, although cancer patients may take a range of drugs, they may not always manage to control their symptoms.

Oncological patients are often treated with a range of drugs (e.g., anticancer, antiemetic, psychopharmaceutical, and analgesic drugs), which increases the risk of drug interactions and adverse effects [16]. The risks of polypharmacy also include an increase in episodes of falls, frailty, hospitalization, postoperative complications, and even higher mortality [16]. All these problems can lead to greater disability and lower patient autonomy, particularly for older patients [17].

This evidence highlights the need for strategies that can control pain and emotional distress in patients undergoing complex oncological processes but that help to decrease polypharmacy as much as possible.

There is evidence that complementary therapies, such as muscle relaxation, are effective in improving the quality of life of patients with cancer [18–26]. However, there is a lack of research on the effect of such therapies on pain and the consumption of psychotropic drugs and analgesics in cancer patients.

Therefore, the aim of this study was to determine whether the practice of Jacobson’s relaxation technique reduced consumption of psychotropic and analgesic drugs in a sample of cancer patients.

## Methods

### Design

This was a multicenter pre–post intervention study. The study was conducted in accordance with the CONSORT guidelines.

### Participants

The study was conducted between November 1, 2014, and October 1, 2015, in the oncological units of 10 Spanish public hospitals. Patient recruitment took place in these units using posters, informative flyers, and information provided to relevant health professionals (oncologists, nurses, and psychologists). Cancer patients exhibiting anxiety, muscular tension, sleeping difficulties, sadness, or anxiety attacks, and who agreed to participate, were recruited. The exclusion criteria were patients exhibiting hallucinations, delirium, or other psychotic symptoms, because the practice of muscle relaxation can lead to potentially unpleasant extracorporeal sensations.

### Data collection

The following participant data were collected: 1) sociodemographic and medical characteristics: medical center, age, gender, marital status, and educational level; 2) oncological process: cancer diagnosis, cancer therapy (chemotherapy, radiotherapy, hormone therapy, biological therapy, and surgery), any side effects of the cancer treatment, cancer pain, and analgesic use; 3) mental health issues: use of anxiolytics, hypnotics, and antidepressants; 4) other variables related to the intervention, such as symptoms motivating inclusion in the study and questions such as “Have you practiced the technique at home?” or “What is the frequency, per week, that you practice the technique?” Participants were also asked whether the technique helped them control the anxiety and pain. Finally, the level of anxiety prior to the session was recorded, together with the degree of relaxation achieved, the degree of concentration during the exercises, the command of the technique, and the level of confidence in its use.

One researcher collected data on the day of the session and once a week during the 4 weeks follow-up via phone communication.

In this study, the effect of the intervention on medication consumption could only be assessed for drugs issued as emergency prescriptions or refills.

### Sample size calculation

The sample size was calculated using EPIDATA version 4.1. As different hospitals were gradually included throughout the study, we estimated the sample size based on an infinite population-based sample. With a 95% confidence level, an expected proportion of 20% of anxiety disorders in the cancer population, and a maximum error of estimation of 5%, the estimated sample size was 246 patients. An expected loss rate of 5% was assumed; therefore, the final estimated sample size was 259 patients.

### Intervention

A guided session was organized for all participants to teach them the abbreviated progressive muscle relaxation training developed by Bernstein and Borkovec [27]. The sessions were conducted individually or in groups, according to the needs of each patient. To reduce possible interexaminer bias and ensure consistency in the selection criteria, all researchers conducting the intervention had been fully trained on the selection criteria, the information provided to participants, the data collection procedures, and the application of the technique. Written guidance on the relaxation sessions was distributed to all researchers. The main researcher was present at the initial treatment intervention at all hospital centers. A pilot test was performed with the initial 30 patients. Patients were seated during the performance of the technique, which took place in comfortably furnished rooms with armchairs, cushions, soothing lighting, and an overall tranquil environment. Each session lasted for approximately 1 h and consisted of an explanation of the main characteristics of the abbreviated Jacobson’s relaxation technique developed by Bernstein and Borkovec [27]; a relaxation session; an opportunity for participants to ask questions; and data collection using a data collection notebook established for this study.

Upon completion of the session, patients were given an information sheet about the intervention. This comprised a brief description of the session, based on text and images, to help them practice the technique at home.

### Ethical considerations

Ethical approval was granted by the corresponding clinical research ethical committee of each center involved in the study [28] (University Hospital of Getafe, 06/26/2014; Puerta del Hierro-Majadahonda, 07/24/2014; Foundation Alcorcon, 11/03/2014; Fuenlabrada, 12/03/2014; Bellvitge, 09/10/2014; Salamanca, 07/18/2014; Navarra, 03/27/2015; Hospital Germans Trias i Pujol, 11/21/2014; and Cantabria 08/01/2014). All data were treated anonymously and confidentially according to the Spanish Personal Data Protection Act [29]. The trial was registered with ISRCTN (trial number 81335752). The clinical trial registration was postponed because the study was registered with the Spanish Agency of Medicine and, therefore, inclusion in the Spanish Registry of Clinical Studies was not required. In addition, the study sponsor considered the study to be a behavioral intervention rather than a clinical trial owing to the lack of drugs, biologics, and devices. All procedures were conducted according to the Declaration of Helsinki [30]. All participants provided written informed consent after having been fully informed about the study aims. Finally, although adverse effects from the practice of this technique have not been reported, it is important to emphasize that these types of techniques are not a substitute for medical treatment.

### Data analysis

The SAS v9.3 software (SAS Institute Inc., Cary, NC, USA) was used for statistical analyses. The level of significance for statistical decision-making was set at 0.05. A descriptive univariate analysis was conducted: for qualitative variables we report percentages and 95% confidence intervals (CI) for each category; and for quantitative variables we report means and standard deviations (SD).

To determine changes in the assessed variables throughout the 4 weeks, we used adjusted models for each assessment according to the week and the initial assessment, and incorporated repeated measures of each subject throughout the entire 4-week period [31]. Logistic regression was used for qualitative variables (“Have you practiced the technique at home?”, “Do you think that the technique is helping you to control the anxiety symptoms?”, “Do you think that the technique is helping you to control the pain?”), and linear regression was used for quantitative variables (anxiety, relaxation, concentration, and mastery of the technique). We calculated estimates and 95% CIs of the mean (or percentage) for each assessment and week, and calculated adjusted contrasts using the Tukey correction to analyze differences in the variables between 2 consecutive weeks.

## Results

**Fig. 1:**
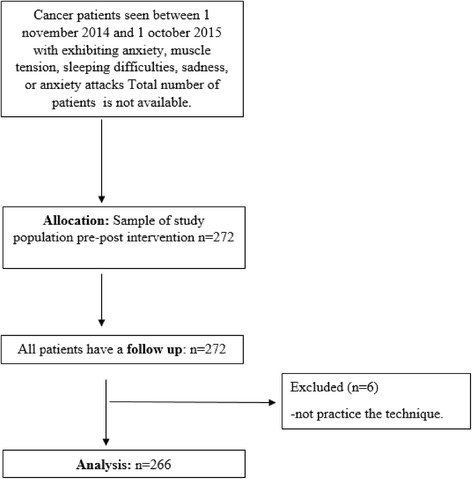
Flowchart of study participants

Initial recruitment comprised 272 patients from the oncological services of the participating hospitals who were experiencing anxiety, satisfied all eligibility criteria, and agreed to participate. Of these, six (2%) were excluded from the analysis, as they failed to practice the technique at home (Fig. 1).

Table 1 describes the sociodemographic characteristics, medical characteristics, and effects of the intervention for patients that practiced the technique at home. The mean age was 52.56 years [SD, 11.33 years], and 76.32% (95% CI, 71.18–81.45) of the sample were women. Up to 96.24% (95% CI, 93.94–98.54) were receiving chemotherapy treatment and 86.84% (95% CI, 82.76–90.92) reported side effects. In total, 46.62% (95% CI, 40.59–52.64) reported pain, for which 100% took an analgesic. Regarding psychotropic drugs, 31.58% (95% CI, 25.97–37.19) took anxiolytics, 12.03% (95% CI, 8.1–15.96) took antidepressants, and 21.80% (95% CI, 16.82–26.79) took hypnotics.

**Table 1:** Sociodemographic, medical characteristics on study participants

	TOTAL		
	Number	Percent	95%CI
Medical center			
University Hospital “Marques de Valdecilla”, Cantabria.	16	6.02	3.14–8.89
“Fundación Alcorcón” Hospital, Madrid.	35	13.16	9.08–17.24
University Hospital of Getafe, Madrid.	17	6.39	3.44–9.34
University Hospital of Fuenlabrada, Madrid.	52	19.55	14.76–24.34
Catalan Institute of Oncology, University Hospital of Bellvitge, Barcelona.	35	13.16	9.08–17..24
Catalan Institute of Oncology, University Hospital “Germans Trias i Pujol”, Barcelona.	45	16.92	12.39–21.44
Hospital “Sierrallana”, Cantabria.	4	1.50	0.03–2.97
Hospital of Navarra, Navarra.	47	17.67	13.06–22.27
Hospital of Salamanca, Salamanca.	5	1.88	0.24–3.52
University Hospital “Puerta de Hierro-Majadahonda”, Madrid.	10	3.76	1.46–6.06
AGE (years) Mean [SD]	52.56	[11.33]	
Gender			
Female	203	76.32	71.18–81.45
Male	63	23.68	18.55–28.82
Marital status			
Married	178	66.92	61.24–72.6
Single	39	14.66	10.39–18.93
Divorced	11	4.14	1.73–6.54
Widowed	10	3.76	1.46–6.06
Separated	11	4.14	1.73–6.54
Domestic partnership	17	6.39	3.44–9.34
Educational level			
Elementary	131	49.25	43.21–55.28
Secondary	87	32.71	27.04–38.37
University	48	18.05	13.4–22.69
Cancer diagnosis			
Lung	31	11.65	7.78–15.52
Digestive	36	13.53	9.4–17.66
Head and neck	5	1.88	0.24–3.52
Gynecological	139	52.26	46.23–58.29
Urinary	7	2.63	0,7–4.56
Hematological malignancies	41	15.41	11.05–19.77
Others	7	2.63	0.7–4.56
Cancer therapy			
Chemotherapy	256	96.24	93.94–98.54
Radiotherapy	121	45.49	39.48–51.5
Hormone therapy	52	19.55	14.76–24.34
Biological therapy	50	18.80	14.08–23.51
Surgery to remove cancer	147	55.26	49.26–61.27
Side effects of cancer treatment			
No	35	13.16	9.08–17.24
Yes	231	86.84	82.76–90.92
Cancer pain			
No	142	53.38	47.36–59.41
Yes	124	46.62	40.59–52.64
Use of analgesics			
No	–	–	
Yes	124	100	–
Use of anxiolytics			
No	182	68.42	62.81–74.03
Yes	84	31.58	25.97–37.19
Use of antidepressants			
No	234	87.97	84.04–91.9
Yes	32	12.03	8.1–15.96
Use of hypnotics			
No	208	78.20	73.21–83.18
Yes	58	21.80	16.82–26.79
Symptoms of inclusion in the study			
Anxiety	261	98.12	96.48–99.76
Insomnia	37	13.91	9.73–18.09
Sadness	15	5.64	2.85–8.42
Muscle tension	3	1.13	0–2.4

*SD* Standard deviation, *CI* Confidence interval

Table 2 shows the changes over the 4 weeks in anxiety, relaxation, concentration, and mastery of the technique, and the tests of fixed effects in the model (using the type III sum of squares test). Changes in the assessed parameters were influenced by the 4-week follow-up as a covariate and by the initial value. The anxiety level prior to the session was positively influenced by the initial value (F(1,1014) = 2464.7; *p* < 0.001) and negatively influenced by the 4-week follow-up (F(3,1014) = 11.66; p < 0.001). In the other models, the value of the assessed parameters was positively influenced by the initial value and by the 4-week follow-up.

**Table 2:** Evolution throughout the 4 weeks for anxiety, relaxation, concentration and mastery over the technique variables

	Number	Mean	95%CI
Level of anxiety prior to the session			
Day of the intervention	266	3.99	3.63–4.34
Week 1	254	3.87	3.54–4.20
Week 2	257	3.52	3.25–3.79
Week 3	254	3.35	3.08–3.63
Week 4	254	3.26	2.98–3.54
Level of relaxation achieved			
Day of the intervention	266	5.19	4.88–5.49
Week 1	254	3.70	3.52–3.88
Week 2	257	5.46	5.28–5.64
Week 3	254	6.12	5.95–6.28
Week 4	254	6.55	6.38–6.72
Level of concentration during the exercises			
Day of the intervention	266	4.11	3.85–4.36
Week 1	254	3.33	3.15–3.50
Week 2	257	4.51	4.32–4.70
Week 3	254	5.17	4.98–5.36
Week 4	254	5.74	5.57–5.92
Mastery over the technique			
Day of the intervention	266	3.28	3.06–3.49
Week 1	254	3.83	3.63–4.03
Week 2	257	4.29	4.07–4.51
Week 3	254	4.98	4.78–5.18
Week 4	254	5.48	5.29–5.67
Confidence in its usefulness			
Day of the intervention	266	5.95	5.73–6.18
Week 1	254	6.20	5.98–6.42
Week 2	257	6.27	6.06–6.48
Week 3	254	6.59	6.39–6.79
Week 4	254	6.86	6.66–7.05

*CI* Confidence Interval

Table 3 highlights the differences in estimates for the parameters evaluated for each of 2 consecutive weeks. This enabled us to follow the changes in each parameter using the estimated difference between 2 consecutive weeks. As the table shows, for all the assessed parameters, there was a statistically significant difference between at least one pair of consecutive weeks. The differences between 2 consecutive weeks were statistically significant for each pair of consecutive weeks for relaxation, concentration, and mastery of the technique.

**Table 3:** Highlights the differences in estimation for the parameters evaluated for each two consecutive weeks

	Comparison		Estimate of the difference			
	Time 1	Time 2	Estimate of the difference	95%CI inferior	95%CI superior	*p* value*
Level of anxiety prior to the session	Week 1	Week 2	0.32	0.03	0.61	0.023
	Week 2	Week 3	0.20	−0.09	0.48	0.296
	Week 3	Week 4	0.10	−0.19	0.39	0.817
Level of relaxation achieved	Week 1	Week 2	−1.75	−2.06	−1.45	<.001
	Week 2	Week 3	−0.66	− 0.97	− 0.36	<.001
	Week 3	Week 4	−0.43	− 0.74	− 0.13	0.001
Level of concentration during the exercises	Week 1	Week 2	−1.17	− 1.47	−0.88	<.001
	Week 2	Week 3	−0.67	−0.97	− 0.38	<.001
	Week 3	Week 4	−0.57	−0.87	− 0.28	<.001
Mastery over the technique	Week 1	Week 2	−0.45	−0.74	− 0.15	<.001
	Week 2	Week 3	−0.70	−1.00	−0.41	<.001
	Week 3	Week 4	−0.50	−0.79	− 0.20	<.001
Confidence in its usefulness	Week 1	Week 2	−0.06	−0.27	0.15	0.865
	Week 2	Week 3	−0.33	−0.54	− 0.12	<.001
	Week 3	Week 4	−0.27	−0.48	− 0.06	0.006

Table 4 shows the consumption reduction for psychotropic and analgesic drugs throughout the 4 weeks of follow-up. After the intervention, a maximum of 14.71% (95% CI, 5.55–23.86) patients did not require anxiolytics after 1 week, and a maximum of 26.47% (95% CI, 15.25–37.69) patients per week required these drugs on fewer occasions.

**Table 4:** Use of psychotropic and analgesic drugs after the intervention

	*N* = 266	Percent	95%CI
ANXIOLYTIC USE	84	31.58	25.81–37.65
Consumption “on demand”	68	80.95	71.96–89.95
Does not require anxiolytics			
Week 1	8	11.76	3.37–20.16
Week 2	9	13.23	4.45–22.03
Week 3	10	14.71	5.55–23.86
Week 4	10	14.71	5.55–23.86
Requires anxiolytic on fewer occasions			
Week 1	18	26.47	15.25–37.69
Week 2	6	8.82	1.35–16.30
Week 3	6	8.82	1.35–16.30
Week 4	6	8.82	1.35–16.30
HYPNOTIC USE	58	21.80	16.65–26.95
Consumption “on demand”	40	68.97	56.20–81.73
Does not require hypnotics			
Week 1	1	2.5	0.06–13.16
Week 2	3	7.5	1.57–20.39
Week 3	4	10	2.79–23.66
Week 4	5	12.5	4.19–26.80
Requires hypnotics on fewer occasions			
Week 1	5	12.5	4.19–26.80
Week 2	6	15	2.68–27.32
Week 3	5	12.5	4.19–26.80
Week 4	4	10	2.79–23.66
USE OF ANALGESICS	124	46.62	40.43–52.80
Emergency prescriptions	52	41.94	32.85–51.02
Does not require analgesia			
Week 1	2	3.85	0.47–13.21
Week 2	2	3.85	0.47–13.21
Week 3	2	3.85	0.47–13.21
Week 4	2	3.85	0.47–13.21
Requires analgesia on fewer occasions			
Week 1	16	30.77	17.26–44.27
Week 2	16	30.77	17.26–44.27
Week 3	16	30.77	17.26–44.27
Week 4	16	30.77	17.26–44.27
Analgesics are more effective			
Week 1	9	17.31	6.06–28.55
Week 2	8	15.38	4.62–26.15
Week 3	8	15.38	4.62–26.15
Week 4	8	15.38	4.62–26.15

*CI* Confidence interval

Finally, Table 5 describes the effects of the intervention for patients who practiced the technique at home. Throughout the 4-week follow-up period, over 95% of patients had practiced the technique at home, and the mean frequency of practice per week was 6.19–6.57. Over 97% of patients each week reported that relaxation had helped them to control anxiety symptoms. Between 21.01% and 22.69 of patients with pain reported an improvement in pain control.

**Table 5:** Effects of intervention on study participants (post intervention)

	TOTAL		
	Number	Percent	95%CI
Have you practiced the technique at home?			
Week 1	254	95.49	92.98–97.99
Week 2	257	96.62	94.43–98.8
Week 3	254	95.49	92.98–97.99
Week 4	254	95.49	92.98–97.99
How many times have you practiced the technique per week?			
Week 1 Mean [SD]	254	6.57 [4.36]	
Week 2 Mean [SD]	257	6.46 [3.62]	
Week 3 Mean [SD]	254	6.24 [3.57]	
Week 4 Mean [SD]	254	6.19 [3.65]	
Do you consider that the technique is helping you to control the symptoms derived from anxiety?			
Week 1	247	97.24	95.22
Week 2	250	97.28	95.28–99.28
Week 3	248	97.64	95.76–99.51
Week 4	248	97.64	95.76–99.51
Do you consider that the technique is helping you to control the pain?			
Week 1	27	22.69	15.13–30.25
Week 2	25	21.01	13.66–28.36
Week 3	26	21.85	14.39–29.31
Week 4	26	21.85	14.39–29.31

*SD* Standard deviation,*CI* Confidence interval

A maximum of 12.5% (95% CI, 4.19–26.80) patients did not require hypnotics after 1 week and a maximum of 15% (95% CI, 2.68–27.32) required them on fewer occasions. Finally, after the intervention, a maximum of 38.5% (95% CI, 0.47–13.21) patients did not require analgesics after 1 week and a maximum of 30.77% (95% CI, 17.26–44.27) required them on fewer occasions. Furthermore, a maximum of 17.31% (95% CI, 6.06–28.55) of patients reported that the analgesics were more effective.

## Discussion

The practice of complementary techniques that induce relaxation can improve the quality of life of patients [18–26], and this can result in a decrease of the consumption of certain drugs for some patients.

Between 97.24% and 97.24% of patients who practiced the technique reported an improvement in anxiety. This led to a reduction in the consumption of anxiolytics in a maximum of 26.47% of patients after 1 week and a maximum of 14.71% of patients after 1 week did not require anxiolytic medication.

These results support previous findings by Beard et al. [23], who found statistically significant differences in pre–post anxiety measurements in groups treated with Reiki and relaxation compared with a control group. In addition, Isa et al. [24, 25] investigated the effect of muscle relaxation on anxiety and stress levels among patients with prostate cancer and reported significant differences at 4 and 6 months.

Other complementary therapies have also demonstrated effectiveness in controlling anxiety. Yoga significantly reduced both initial and final anxiety levels (measured using the State–Trait Anxiety Inventory) after an 8-week intervention [21]. A study of patients with breast cancer showed a significant improvement on Hospital Anxiety and Depression Scale scores after weekly acupuncture treatment [20]. The effect of muscle relaxation on the control of anxiety has also been studied in non-oncological patients, such as pregnant women [32], patients with dental anxiety [33], pulmonary hypertension patients [34] and schizophrenia patients [35].

None of these studies investigated the consumption of psychotropic drugs. Our findings suggest that complementary therapies could help patients to reduce the consumption of emergency or refill prescription drugs, as was the case for the patients in our sample.

Despite the fact that patients reported improvements in pain (21.01%–22.69%), this effect had a moderate impact on analgesic consumption (up to 3.85% of participants did not require these and up to 30.77% required them on fewer occasions). Notwithstanding, some patients reported that the analgesics were more effective (17.31%). Neither Beard et al. [23] nor Andersen et al. [19] (who studied muscle relaxation in cancer patients) investigated pain; however, the literature suggests that techniques that involve relaxation, such as music therapy and yoga, can improve pain control [21, 35–41].

Patients experienced positive changes in all the parameters studied throughout the 1-month follow-up. As patients continued to practice the technique, they experienced an increase in relaxation, concentration, mastery of the technique, and confidence in its usefulness, at least during the first month. Furthermore, anxiety levels prior to the relaxation session decreased over the weeks, suggesting a greater control over anxiety. Our relaxation protocol only included a 1-month follow-up, and we found improvements after the first week. Other similar studies [19, 23–25] have lasted for several weeks or months. At present, there is a lack of research on the use of this technique with similar follow-up periods in cancer patients. Thus, future studies need to assess the long-term effect of the intervention.

One of the main differences between our intervention and those used in previous studies is the simplicity of the training. Patients only required a 1-h session to learn the technique, which can be performed in a few minutes in a seated position. Furthermore, although the technique is more effective if practiced in a silent environment with dimmed light, it can be performed anywhere. Therefore, this protocol could be implemented in different hospitals, as it does not require complex resources.

Although complementary techniques are increasingly used in oncology, there are not enough studies to compare our results to; therefore, further studies on this technique are required.

This study has several strengths, such as the use of a large sample from different hospitals (i.e., this was a multicenter study) and the training of staff in the technique. However, there are potential limitations. The lack of a control group means that we cannot confirm that the results derived solely from the intervention. However, the inclusion of control/placebo groups in studies of cancer patients is a challenging procedure from an ethical perspective. In addition, patients were recruited in hospitals using informative flyers, posters, and direct information provided by health professionals caring for the patients. Therefore, we cannot confirm exactly how many prospective participants received this information. Finally, we only evaluated short-term effects of the intervention, so are unable to determine the long-term effects. Nevertheless, as the mortality rate of several cancer types included in the current study was high, this would be challenging data to obtain in future studies.

## Conclusions

The practice of abbreviated Jacobson’s relaxation technique developed by Bernstein and Borkovec [27] helped to reduce the consumption of psychotropic and analgesic drugs. Patients experienced positive changes in all the evaluated parameters, at least during the 1-month follow-up. To confirm our findings, additional long-term intervention studies are needed that include control groups.
